# Aztreonam–avibactam for the treatment of serious infections caused by metallo-β-lactamase-producing Gram-negative pathogens: a Phase 3 randomized trial (ASSEMBLE)

**DOI:** 10.1093/jacamr/dlaf131

**Published:** 2025-07-28

**Authors:** George L Daikos, José Miguel Cisneros, Yehuda Carmeli, Minggui Wang, Chee Loon Leong, Konstantinos Pontikis, Anastasia Anderzhanova, Simin Florescu, Roman Kozlov, Eduardo Rodriguez-Noriega, Mina Psichogiou, Pinyo Rattanaumpawan, Anca Streinu-Cercel, Venkatasubramanian Ramasubramanian, Francis F Arhin, Halley Rogers, Michele Wible, Joanne Leaney, Daria Jacobson, Rienk Pypstra, Joseph W Chow

**Affiliations:** Department of Medicine, National and Kapodistrian University of Athens, 115-27 Athens, Greece; Virgen del Rocío University Hospital-IBiS, CIBERINFEC, Seville, Spain; The National Center for Antibiotic Resistance and Infection Control, Tel Aviv Sourasky Medical Center, Tel Aviv, Israel; Institute of Antibiotics, Huashan Hospital, Fudan University, Shanghai, China; Key Laboratory of Clinical Pharmacology of Antibiotics, National Heath Commission of People’s Republic of China, Shanghai, China; Department of Medicine, Hospital Kuala Lumpur, Kuala Lumpur, Malaysia; 1st Department of Respiratory Diseases, General and Chest Diseases Hospital ‘Sotiria’, Athens, Greece; Clinical Pharmacology, SBHI of the City of Moscow ‘N.I.Pirogov City Clinical Hospital #1’, Moscow, Russian Federation; Infectious Diseases Department, Clinical Hospital of Infectious and Tropical Diseases, ‘Dr. Victor Babeş ’, Bucharest, Romania; Infectious Diseases Department, Carol Davila University of Medicine and Pharmacy, Bucharest, Romania; Smolensk State Medical University, Ministry of Health of the RF, Smolensk Regional Clinical Hospital, SRI of Antimicrobial Chemotherapy 46A, Smolensk, Russian Federation; Infectious Diseases, Hospital Civil de Guadalajara ‘Fray Antonio Alcalde’, Guadalajara, Mexico; 1st Department of Internal Medicine, General Hospital of Athens ‘Laiko’, Athens, Greece; Faculty of Medicine Siriraj Hospital, Mahidol University, Bangkok, Thailand; Infectious Diseases Department, Carol Davila University of Medicine and Pharmacy, Bucharest, Romania; Infectious Diseases Department, Institutul National de Boli Infecţioase ‘Prof. Dr. Matei Balş’, Bucharest, Romania; Infectious Diseases, Apollo Hospitals, Chennai, India; Developmental Microbiology, Pfizer Inc., Kirkland, QC, Canada; Clinical Development & Operations, Pfizer Inc., New York, NY, USA; Global Product Development, Pfizer Inc., Collegeville, PA, USA; Global Research & Development, Pfizer Inc., Sandwich, Kent, UK; Oncology Division, Pfizer Inc., Herzliya Pituach, Israel; Clinical Development & Operations, Pfizer Inc., New York, NY, USA; Global Product Development, Pfizer Inc., Collegeville, PA, USA

## Abstract

**Background:**

The Phase 3 ASSEMBLE study investigated aztreonam–avibactam versus best available therapy (BAT) for treatment of complicated intra-abdominal infection (cIAI), complicated urinary tract infection (cUTI), hospital-acquired/ventilator-associated pneumonia (HAP/VAP) or bloodstream infection (BSI) caused by confirmed MBL-producing multidrug-resistant pathogens.

**Methods:**

This prospective, multicentre, randomized, open-label, central assessor-blinded study randomized hospitalized adults 2:1 to aztreonam–avibactam [+ metronidazole (cIAI)] or BAT for 5–14 (cIAI, cUTI and BSI) or 7–14 (HAP/VAP) days. Primary endpoint was clinical cure at test-of-cure (TOC) visit on Day 28  ±  3 [microbiological ITT (micro-ITT) analysis set]. Secondary endpoints included microbiological response at TOC, 28-day mortality and safety. No formal hypothesis testing was planned.

**Results:**

Fifteen patients were randomized [aztreonam–avibactam, *n* = 12; BAT, *n* = 3 (ITT and micro-ITT analysis sets)]. Most frequent baseline pathogens were Enterobacterales; *Klebsiella pneumoniae* was most common [aztreonam–avibactam, 6/12 (50%); BAT, 2/3 (67%)]. MBL subtypes/variants identified in the aztreonam–avibactam group were NDM-1 (*n* = 7), NDM-5 (*n* = 3), VIM-2 (*n* = 2) and L1 (*n* = 3); and for BAT were NDM-1 (*n* = 2) and NDM-5 (*n* = 1). Clinical cure rates at TOC were 5/12 (42%) for aztreonam–avibactam and 0/3 (0%) for BAT. Per-patient microbiological responses were generally consistent with clinical responses. Twenty-eight-day all-cause mortality rates for aztreonam–avibactam and BAT were 1/12 (8%) and 1/3 (33%), respectively. Aztreonam–avibactam was generally well-tolerated, with no treatment-related serious adverse events.

**Conclusions:**

These Phase 3 data provide support for aztreonam–avibactam as a potential therapeutic option for difficult-to-treat infections caused by MBL-producing Gram-negative bacteria.

## Introduction

MDR Gram-negative Enterobacterales, including those producing Ambler Class B MBLs, represent a significant threat to patients and healthcare systems, as treatment options are extremely limited.^1,2^

Aztreonam–avibactam is approved in Europe for the treatment of adults with complicated intra-abdominal infections (cIAIs); hospital-acquired pneumonia (HAP), including ventilator-associated pneumonia (VAP); and complicated urinary tract infections (cUTIs), including pyelonephritis; and for infections due to aerobic Gram-negative organisms with limited treatment options.^3^ Aztreonam–avibactam received FDA approval in 2025 for treatment (with metronidazole) of cIAI in adult patients with limited treatment options.^4^ Aztreonam–avibactam has potent *in vitro* activity against MDR Enterobacterales, including strains that produce extended-spectrum β-lactamases, serine carbapenemases and MBLs.^5–12^

The Phase 3 REVISIT study showed aztreonam–avibactam to be effective in treating patients with cIAI or HAP/VAP, including severely ill patients and those with prior treatment failure.^13^

We report results of the ASSEMBLE study evaluating the efficacy and safety of aztreonam–avibactam versus best available therapy (BAT) in the treatment of serious infections caused by MBL-producing MDR pathogens for which there are limited or no treatment options.

## Methods

### Ethics

The ASSEMBLE (NCT03580044; EudraCT 2017-004544-38) study was undertaken in accordance with Good Clinical Practice guidelines, the Declaration of Helsinki and national and institutional standards. Written informed consent was obtained from patients before screening. Independent ethics committees and/or institutional review boards approved the final study protocol [details provided in the Supplementary Methods (available as Supplementary data at *JAC-AMR* Online)].

### Study design and patients

ASSEMBLE was a Phase 3, prospective, randomized, open-label, parallel group, multinational, comparative study. Detailed methods including patient eligibility criteria, study treatments, assessments, analysis sets and statistical methods are included in the Supplementary Methods and Figure S1. In brief, hospitalized adults with a confirmed diagnosis of cIAI, cUTI (with quantitative urine culture), bloodstream infection (BSI) or HAP/VAP and confirmed MBL-positive Gram-negative bacteria were randomized 2:1 to aztreonam–avibactam (plus metronidazole for cIAI) or BAT according to a central randomization schedule and stratified by infection type.

Genotypic confirmation of at least one MBL-positive Gram-negative pathogen within 7 days before screening was required for study inclusion eligibility. Aztreonam–avibactam and meropenem susceptibility results were available to clinicians through local disk susceptibility testing via central laboratory-provided disks.

The primary efficacy endpoint was proportion of patients with clinical cure at the test-of-cure (TOC) visit [microbiological ITT (micro-ITT) analysis set] as assessed by a treatment-blinded adjudication committee. Secondary endpoints included clinical response at TOC (microbiologically evaluable analysis set); per-patient and per-pathogen microbiological response at TOC (micro-ITT analysis set); 28-day all-cause mortality (ITT analysis set); and safety.

## Results

### Patients

The study was conducted between December 2020 and January 2023. Recruitment of patients with serious infections caused by MBL-producing Gram-negative bacteria proved to be more challenging than anticipated, and the study was terminated prior to reaching the planned enrolment of ∼60 patients. In total, 15 patients were randomized and included in the ITT and micro-ITT analysis sets (aztreonam–avibactam, *n* = 12; BAT, *n* = 3) (Figure S1). One patient assigned to BAT was randomized but did not receive study treatment (due to patient decision); the safety analysis set therefore included 14 patients. Patients’ baseline characteristics are summarized in Table 1.

**Table 1.** Patients’ demographic and baseline characteristics (micro-ITT analysis set)

	Aztreonam–avibactam (*n* = 12)	Best available therapy (*n* = 3)
Age, mean (SD), years	56.6 (17.1)	65.7 (6.7)
Sex, *n* (%)		
Male	8 (66.7)	1 (33.3)
Female	4 (33.3)	2 (66.7)
Race, *n* (%)		
White	5 (41.7)	2 (66.7)
Asian	6 (50.0)	1 (33.3)
American Indian or Alaska Native	1 (8.3)	0
Ethnicity, *n* (%)		
Hispanic or Latino	1 (8.3)	0
Not Hispanic or Latino	11 (91.7)	3 (100.0)
Type of infection, *n* (%)		
cIAI	2 (16.7)	0
HAP/VAP	3 (25.0)	1 (33.3)
cUTI	3 (25.0)	1 (33.3)
BSI	4 (33.3)	1 (33.3)
BMI, mean (SD), kg/m^2^	27.4 (6.1)	23.6 (2.7)
CrCl, median (range), mL/min	73.5 (15.0, 238.0)	104.0 (54.0, 289.0)
Renal function group, *n* (%)		
CrCl >15 to ≤30 mL/min	1 (8.3)	0
CrCl >30 to ≤50 mL/min	1 (8.3)	0
CrCl >50 to ≤150 mL/min	7 (58.3)	2 (66.7)
CrCl >150 mL/min	3 (25.0)	1 (33.3)
APACHE II score, median (range)	13.0 (0.0, 25.0)	8.0 (6.0, 22.0)
APACHE II score category, *n* (%)		
≤10	4 (33.3)	2 (66.7)
>10	8 (66.7)	1 (33.3)
Source of pneumonia, *n* (%)^a^		
Hospital	1 (8.3)	0
Ventilator	2 (16.7)	1 (33.3)
Mechanical ventilation at baseline, *n* (%)^a^	2 (16.7)	1 (33.3)
Previous treatment failure, *n* (%)	8 (66.7)	2 (66.7)
Patients with at least one Gram-negative pathogen, *n* (%)	12 (100.0)	3 (100.0)
Enterobacterales	10 (83.3)	3 (100.0)
*Pseudomonas aeruginosa*	2 (16.7)	0
*Stenotrophomonas maltophilia*	3 (25.0)	0
Infection composition, *n* (%)		
Monomicrobial	6 (50.0)	3 (100.0)
Polymicrobial	6 (50.0)	0
Pathogen resistance subtypes, *n* (%)		
ESBL-positive	9 (75.0)	3 (100.0)
Serine carbapenemase-positive	3 (25.0)	0
MBL-positive^b^	12 (100.0)	3 (100.0)
NDM-1	6 (50.0)	2 (66.7)
NDM-5	3 (25.0)	1 (33.3)
VIM-2	2 (16.7)	0
L1	3 (25.0)	0

^a^Mechanical ventilation and source of acquired pneumonia assessed only for HAP/VAP patients.

^b^Three patients (all in the aztreonam–avibactam group) had two MBL-producing pathogens, including one patient with two NDM-1 MBL pathogens.

APACHE, Acute Physiology and Chronic Health Evaluation; BMI, body mass index; BSI, bloodstream infection; cIAI, complicated intra-abdominal infection; CrCl, creatinine clearance; cUTI, complicated urinary tract infection; ESBL, extended-spectrum β-lactamase; HAP, hospital-acquired pneumonia; MBL, metallo-β-lactamase; micro-ITT, microbiological intention-to-treat; NDM, New Delhi MBL; SD, standard deviation; VAP, ventilator-associated pneumonia; VIM, Verona integron-encoded MBL.

### Baseline pathogens

All randomized patients had at least one MBL-positive Gram-negative pathogen identified at baseline (Table 1); 21 baseline pathogens were identified overall, including 20 aerobic Gram-negative pathogens (Table S1). The most frequently isolated baseline pathogens were Enterobacterales, of which *Klebsiella pneumoniae* was the most common [aztreonam–avibactam, *n* = 6 (50%); BAT, *n* = 2 (67%)]. See Supplementary Results for more detail on baseline pathogens and their resistance characteristics.

All baseline Enterobacterales had aztreonam–avibactam MIC  ≤ 4 mg/L (Table S2), except for one NDM-5-producing *Escherichia coli* with MIC of 8 mg/L in a patient with polymicrobial cIAI (resistant to aztreonam–avibactam based on EUCAST interpretive criteria).^14^

### Efficacy

Individual patient-level data including baseline patient and pathogen characteristics, treatment duration, clinical and microbiological responses at TOC, 28-day mortality and patient narratives are shown in Table S2. Adjudicated clinical cure rates at TOC in the micro-ITT analysis set (primary efficacy endpoint) were 5/12 (42%) for aztreonam–avibactam and 0/3 (0%) for BAT (Table 2). Clinical cure rates as assessed by the investigator were 6/12 (50%) for aztreonam–avibactam and 0/3 (0%) for BAT. Adjudicated clinical cure rates in the microbiologically evaluable analysis set at TOC were consistent with the primary analysis (Table S3). Additional description is provided in the Supplementary Results.

**Table 2.** Adjudicated clinical responses, and microbiological responses, at TOC (micro-ITT analysis set)

	*n* (%)	Aztreonam–avibactam (*n* = 12)	Best available therapy (*n* = 3)
Adjudicated clinical response^a^			
All patients	Cure [95% CI]	5 (41.7) [18.0, 68.8]	0
	Failure	5 (41.7)	1 (33.3)
	Indeterminate	2 (16.7)	2 (66.7)
cIAI	*n*	2	0
	Cure	0	—
	Failure	2 (100.0)	—
	Indeterminate	0	—
HAP/VAP	*n*	3	1
	Cure	0	0
	Failure	1 (33.3)	0
	Indeterminate	2 (66.7)	1 (100.0)
cUTI	*n*	3	1
	Cure	2 (66.7)	0
	Failure	1 (33.3)	0
	Indeterminate	0	1 (100.0)
BSI	*n*	4	1
	Cure	3 (75.0)	0
	Failure	1 (25.0)	1 (100.0)
	Indeterminate	0	0
Microbiological response^b^			
All patients	*n*	10	1
	Favourable	6 (60.0)	0 (0)
	Unfavourable	4 (40.0)	1 (100.0)
cIAI	*n*	2	0
	Favourable	0	—
	Unfavourable	2 (100.0)	*—*
HAP/VAP	*n*	1	0
	Favourable	0	*—*
	Unfavourable	1 (100.0)	*—*
cUTI	*n*	3	0
	Favourable	3 (100.0)	—
	Unfavourable	0	—
BSI	*n*	4	1
	Favourable	3 (75.0)	0

Percentages are based on the total number of patients in the treatment arm (*n*).

Two-sided 95% CI calculated using Jeffrey’s method.

^a^Adjudicated clinical response at TOC was the primary efficacy outcome measure. Clinical cure was defined as improvement in baseline signs and symptoms such that no further antimicrobial treatment for the index infection was required, and for patients with cIAI, no unplanned drainage or surgical intervention was necessary since the initial procedure.

^b^Patients with an indeterminate microbiological response were excluded from the analysis of microbiological response; denominators are based on numbers of evaluable patients. Favourable microbiological response was defined as eradication or presumed eradication of the baseline pathogen(s).

BSI, bloodstream infection; CI, confidence interval; cIAI, complicated intra-abdominal infection; cUTI, complicated urinary tract infection; HAP, hospital-acquired pneumonia; micro-ITT, microbiological ITT; n/a, not applicable; TOC, test-of-cure; VAP, ventilator-associated pneumonia.

Per-patient microbiological responses at TOC were generally consistent with clinical responses (Table 2). Favourable per-pathogen microbiological responses at TOC (aztreonam–avibactam group) were 6/12 (50%) for Enterobacterales, 0/2 for *Pseudomonas aeruginosa*, 1/3 (33%) for *Stenotrophomonas maltophilia* and 0/1 for *Enterococcus faecium* (Table S4).

All-cause 28-day mortality rates were 1/12 (8%) for aztreonam–avibactam and 1/3 (33%) for BAT (Table S5). Both deaths occurred in patients with HAP/VAP and neither was considered treatment-related. Another patient with BSI (aztreonam–avibactam group) died on study day 31.

### Safety

All-cause adverse events (AEs) were reported in 11/12 (92%) of patients in the aztreonam–avibactam group and in both patients (100%) in the BAT group (Tables S6 and S7). Treatment-related AEs occurred in 2/12 (17%) patients in the aztreonam–avibactam group (frequent bowel movements and chest discomfort in one patient and increased alanine aminotransferase, aspartate aminotransferase, blood alkaline phosphatase and GGT in another patient) and 1/2 (50%) of patients in the BAT group (acute kidney injury); all were mild or moderate in intensity.

Serious AEs (SAEs) were reported in 5/12 (42%) and 2/2 patients in the aztreonam–avibactam and BAT groups, respectively (Table S8). One SAE (acute kidney injury) in the BAT group was considered treatment-related (no treatment-related SAEs occurred in the aztreonam–avibactam group).

Five liver disorder AEs (all non-severe) were reported in 2/12 (17%) patients in the aztreonam–avibactam group (0% in the BAT group). There were no confirmed Hy’s Law cases. Other AEs of special interest are described in Supplementary Results.

## Discussion

In this study, aztreonam–avibactam (± metronidazole) was effective in treating patients with difficult-to-treat infections caused by confirmed MBL-producing Gram-negative pathogens. Overall, 5/12 (42%) aztreonam–avibactam-treated patients had adjudicated clinical cure at TOC. Microbiological responses broadly mirrored clinical responses, with 6/12 (50%) patients having a favourable microbiological response at TOC. In the BAT group, 0/3 (0%) patients were cured at TOC and 0/3 (0%) had a favourable microbiological response. Given the small numbers of patients, the estimates of clinical cure rate include wide confidence intervals and caution is warranted in interpreting the findings.

The results from ASSEMBLE, the second of two prospective, active-comparator, Phase 3 clinical studies of aztreonam–avibactam (alongside REVISIT in patients with cIAI or HAP/VAP13 and the Phase 2a REJUVENATE trial^15^), supported the regulatory approval of the combination as a treatment option for patients with serious infections with limited or no treatment options, including those caused by MBL-producing Gram-negative bacteria.

The number of patients randomized in ASSEMBLE was lower than planned because of challenges in recruitment of patients with MBL-producing Gram-negative bacteria, exacerbated by the operational challenges of conducting the study during the global COVID-19 pandemic. The small number of patients actually randomized did not allow for the planned 2:1 ratio between treatment groups due to stratification by infection type. Moreover, of the three patients randomized to BAT, one did not receive study treatment and the remaining two received no more than 5 days of study treatment. Nonetheless, the observed clinical and microbiological response outcomes are generally as expected for these patient populations with serious infections, including a high proportion of patients with prior antibiotic treatment failure.

In REVISIT, which included a small proportion of patients with MBL-positive pathogens, overall clinical cure rates at TOC with aztreonam–avibactam [93/282 (68%)] were similar to those in patients treated with meropenem  ±  colistin [92/140 (66%)]; cure rates for those with MBL-positive pathogens were 2/7 (29%) for aztreonam–avibactam and 2/3 (67%) for meropenem ± colistin (micro-ITT analysis set).^13^

In both ASSEMBLE and REVISIT, aztreonam–avibactam was generally well tolerated, with no new safety findings compared with aztreonam alone. No aztreonam–avibactam-treated patients experienced a treatment-related SAE in either study.

Important limitations of the current study include the small sample size, with numbers in the BAT group particularly low. The descriptive (rather than hypothesis testing) design also represents a limitation of the study. The ASSEMBLE and REVISIT studies were not designed for inferential testing of efficacy; nonetheless, they do provide randomized, assessor-blinded descriptive efficacy data and contribute further to the safety database. The streamlined development approach for aztreonam–avibactam has been confirmed by the EMA and FDA,^16^ and the study design was developed with input from both agencies. A further limitation was the open-label design, which was implemented due to practical issues in maintaining treatment blinding with differing aztreonam–avibactam and BAT administration schedules. However, clinical outcomes were assessed by an independent adjudication committee blinded to treatment assignment, with the aim of unbiased and objective adjudication of efficacy assessments. Finally, the efficacy results may be confounded by the high rates of prior antibiotic treatment failure.

In summary, the results from ASSEMBLE provide valuable insights into the clinical effectiveness and safety of aztreonam–avibactam, supporting a potential role for this combination in the management of difficult-to-treat infections caused by MBL-producing Gram-negative bacteria.

## Supplementary Material

dlaf131_Supplementary_Data

## Data Availability

Upon request, and subject to certain criteria, conditions and exceptions (see https://www.pfizer.com/science/clinical-trials/trial-data-and-results for more information), Pfizer will provide access to individual de-identified participant data from Pfizer-sponsored global interventional clinical studies conducted for medicines, vaccines, and medical devices (1) for indications that have been approved in the US and/or EU or (2) in programmes that have been terminated (i.e. development for all indications has been discontinued). Pfizer will also consider requests for the protocol, data dictionary, and statistical analysis plan. Data may be requested from Pfizer trials 24 months after study completion. The de-identified participant data will be made available to researchers whose proposals meet the research criteria and other conditions, and for which an exception does not apply, via a secure portal. To gain access, data requestors must enter into a data access agreement with Pfizer.

## Transparency declarations

G.L.D. is an advisor and consultant for and has received honoraria from Pfizer; and has received honoraria from MSD and Viatris. Y.C. is an advisor for and has received grant support and honoraria from Pfizer, Qpex, Roche and Merck. K.P. has received honoraria from Pfizer, MSD and Menarini. M.P. has received honoraria from Pfizer and GSK. V.R. has received travel grants and honoraria from Pfizer, GSK, Wockhardt, Cipla and Thermo Fisher. F.F.A., R.P. and J.W.C. are former employees of Pfizer. H.R., M. Wible, J.L. and D.J. are employees of and shareholders in Pfizer. J.M.C., M. Wang, C.L.L., A.A., S.F., R.K., E.R.N., P.R. and A.S.-C. have no conflicts of interest to declare.

## Supplementary data

Figure S1 and Tables S1 to S5 are available as Supplementary data at *JAC-AMR*  Online.
