# Supplementary material for: Aztreonam–avibactam for the treatment of serious infections caused by metallo-β-lactamase-producing Gram-negative pathogens: a Phase 3 randomized trial (ASSEMBLE)
Source: JAC Antimicrob Resist. 2025 Jul 28;7(4):dlaf131. doi: 10.1093/jacamr/dlaf131 (PMC12301880; doi:10.1093/jacamr/dlaf131)
Supplement: dlaf131_Supplementary_Data [file dlaf131_supplementary_data.docx]

Supplementary data

Aztreonam-avibactam for the treatment of serious infections caused by metallo-β-lactamase-producing Gram-negative pathogens: a Phase 3 randomized trial (ASSEMBLE)

George L. Daikos^1*^, José Miguel Cisneros^2^, Yehuda Carmeli^3^, Minggui Wang^4^, Chee Loon Leong^5^, Konstantinos Pontikis^6^, Anastasia Anderzhanova^7^, Simin Florescu^8^, Roman Kozlov^9^, Eduardo Rodriguez-Noriega^10^, Mina Psichogiou^11^, Pinyo Rattanaumpawan^12^, Anca Streinu-Cercel^13^, Venkatasubramanian Ramasubramanian^14^, Francis F. Arhin^15^, Halley Rogers^16^, Michele Wible^17^, Joanne Leaney^18^, Daria Jacobson^19^, Rienk Pypstra^16†^, Joseph W. Chow^17^,

^1^National and Kapodistrian University of Athens, Athens, Greece; ^2^Virgen del Rocío University Hospital-IBiS, CIBERINFEC, Seville, Spain; ^3^The National Center for Antibiotic Resistance and Infection Control, Tel Aviv Sourasky Medical Center, Tel Aviv, Israel; ^4^Institute of Antibiotics, Huashan Hospital, Fudan University, Shanghai, and Key Laboratory of Clinical Pharmacology of Antibiotics, National Heath Commission of People’s Republic of China, Shanghai, China; ^5^Hospital Kuala Lumpur, Kuala Lumpur, Malaysia; ^6^General and Chest Diseases Hospital “Sotiria”, Athens, Greece; ^7^SBHI of the City of Moscow “N.I.Pirogov City Clinical Hospital #1”, Moscow, Russian Federation; ^8^Clinical Hospital of Infectious and Tropical Diseases, "Dr. Victor Babeş", Bucharest, Romania; and Carol Davila University of Medicine and Pharmacy, Bucharest, Romania; ^9^Smolensk State Medical University, Ministry of Health of the RF, Smolensk Regional Clinical Hospital, SRI of Antimicrobial Chemotherapy 46A, Smolensk, Russian Federation; ^10^Hospital Civil de Guadalajara "Fray Antonio Alcalde", Guadalajara, Mexico; ^11^General Hospital of Athens "Laiko", Athens, Greece; ^12^Faculty of Medicine Siriraj Hospital, Mahidol University, Bangkok, Thailand; ^13^Institutul National de Boli Infecţioase “Prof. Dr. Matei Balş”, Bucharest, Romania, and Carol Davila Medicine and Pharmacy University, Bucharest, Romania; ^14^Apollo Hospitals, Chennai, India; ^15^Pfizer Inc., Kirkland, QC, Canada; ^16^Pfizer Inc., New York, NY, USA; ^17^Pfizer Inc., Collegeville, PA, USA; ^18^Pfizer Inc., Sandwich, Kent, UK; ^19^Pfizer Inc., Herzliya Pituach, Israel

**^†^**Current affiliation: tranScrip Ltd, Wokingham, UK

**Supplementary methods**

## Ethics committee approval of the study protocol

| **Country** | **IEC/IRB** |
| --- | --- |
| China | Huashan Hospital Fudan University Ethics Committee  NO.12 Middle Urumqi Road  Shanghai, SHANGHAI 200040  CHINA |
|  | Ethics Committee of The First Affiliated Hospital of Shantou University Medical College  No.57 Changping Road  Shantou, GUANGDONG 515041  CHINA |
|  | Ethics Committee of Baotou Central Hospital  Number 61 Huancheng Road, Donghe District  Baotou, INNER MONGOLIA AUTONOMOUS REGION 014000  CHINA |
| Greece | National Ethics Committee, Ministry of Health  284 Mesogeion Avenue, Cholargos  Athens, 15562  GREECE |
| India | Institutional Ethics Committee-Clinical Studies  21 Greams Lane, Off Greams Road,Apollo Hospitals Enterprise Limited  Chennai, TAMILNADU 600006  INDIA |
| Malaysia | Medical Research and Ethics Committee  National Institute of Health, Ministry of Health Malaysia  Block A, Level 2, No. 1, Jalan Setia Murni U13/52, Seksyen U13, Setia Alam  Shah Alam, SELANGOR 40170  MALAYSIA |
| Mexico | Comite de Etica en Investigacion del Hospital Civil de Guadalajara Fray Antonio Alcalde (Inactive  IRB/IEC)  Calle Hospital 278, Colonia Centro  Guadalajara, JALISCO 44280  MEXICO |
|  | Comite de Investigacion Antiguo Hospital Civil de Guadalajara “Fray Antonio Alcalde”  Coronel Calderón No. 777, Col. El Retiro  Guadalajara, JALISCO 44280  MEXICO |
| The Philippines | Davao Doctors Hospital Research Ethics Committee  118 E. Quirino Ave.  Davao City, 8000  PHILIPPINES |
| Romania | Comisia Nationala de Bioetica a Medicamentului si a Dispozitivelor Medicale  Sos. Stefan cel Mare nr. 19-21, sector 2  Bucuresti, 020125  ROMANIA |
| Russian Federation | IEC of FSBEI of HE “Smolensk State Medical University” of the Ministry of Health of the RF  27, pr. Gagarina  Smolensk, 214018  RUSSIAN FEDERATION |
|  |  |
|  | IEC of FSBEI of HE “Smolensk State Medical University” of the Ministry of Health of the RF  28, Krupskoi ul.  Smolensk, 214019  RUSSIAN FEDERATION |
|  | Independent Interdisciplinary Committee on Ethical Review for Clinical Studies  51, Leningradskiy prospect  Moscow, 125468  RUSSIAN FEDERATION |
|  | EC of SBHI of the city of Moscow "N.I.Pirogov City Clinical Hospital # 1"  of the Department of Healthcare of the City of Moscow  8, Leninskiy prospect  Moscow, 119049  RUSSIAN FEDERATION |
| Thailand | Siriraj Institutional Review Board  2 Wang Lang Road, Siriraj  Bangkoknoi, BANGKOK 10700  THAILAND |

## Study design

The study consisted of a screening visit, a baseline visit on day 1 of study treatment, daily treatment phase visits (day 2 up to day 14), an end of treatment (EOT) visit within 24 hours after the last infusion, a test-of-cure (TOC) visit on day 28 ± 3, and a late follow-up visit on day 45 ± 3 (Figure S1).

**Figure S1. Study design**


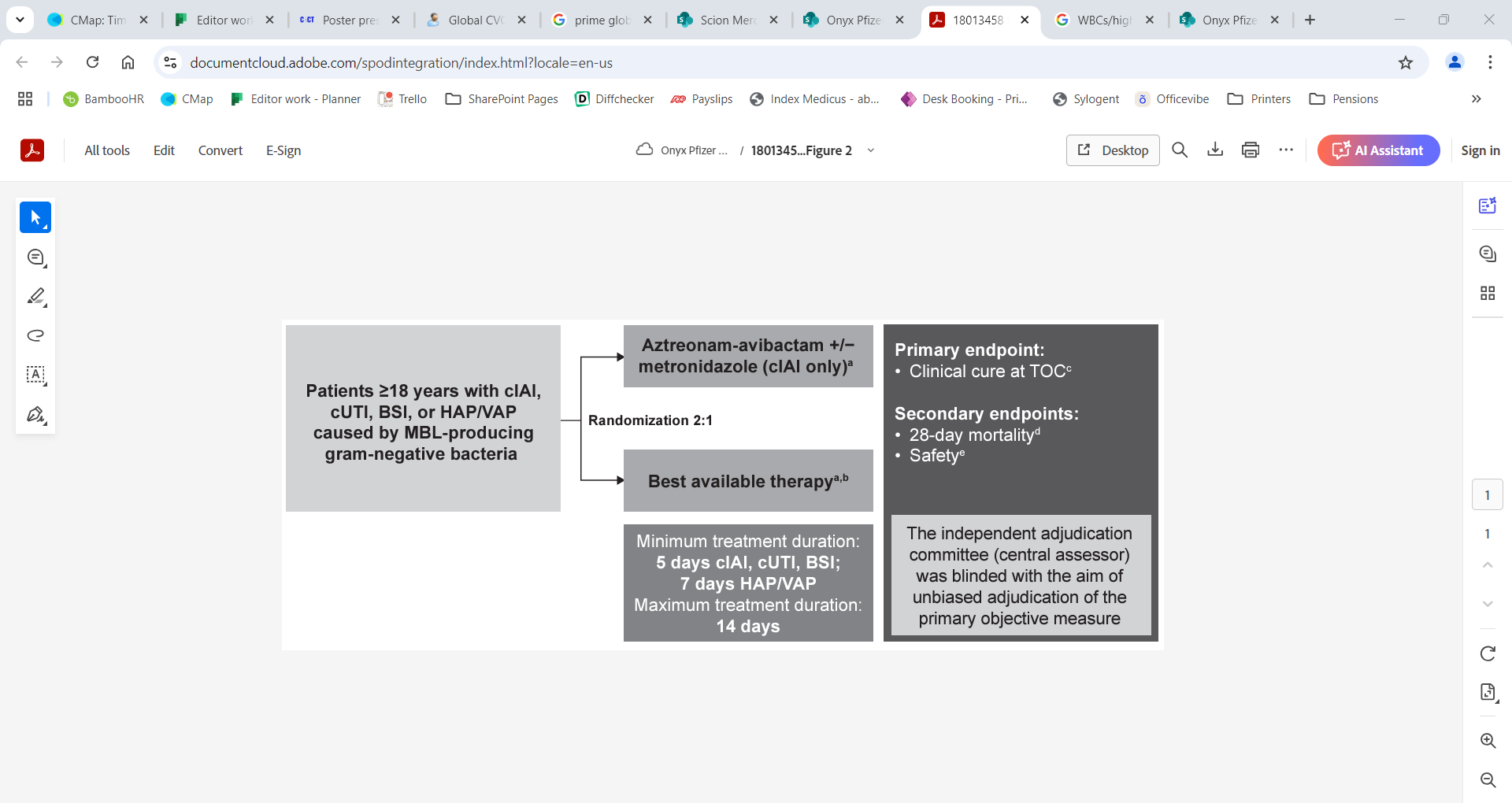


^a^If gram-positive pathogen(s) were suspected or confirmed, vancomycin, linezolid or daptomycin (cIAI only) could be given at the investigator’s discretion. In patients with HAP/VAP and suspected or confirmed *Pseudomonas aeruginosa* infection, an optional intravenous aminoglycoside could be given.
^b^Based on investigative site practice and local epidemiology.  ^c^Micro-ITT analysis set; TOC visit carried out on day 28 (± 3 days).
^d^ITT analysis set.
^e^Safety analysis set.
BSI, bloodstream infection; cIAI, complicated intra-abdominal infection; cUTI, complicated urinary tract infection; HAP, hospital-acquired pneumonia; MBL, metallo-β-lactamases; micro-ITT, microbiological ITT; TOC, test‑of‑cure; VAP, ventilator-associated pneumonia.

Given the limited treatment options for metallo-β-lactamase (MBL)-positive pathogens that may differ between sites due to differing susceptibility patterns, the comparator treatment (best available therapy [BAT]) was selected by investigators based on their study site standard practice and local pathogen resistance epidemiology. The study was therefore open-label based on the resultant impracticalities for double-blinding.

Patients were enrolled at sites in China, Greece, India, Malaysia, Mexico, the Philippines, Romania, Thailand and the Russian Federation.

# Patient eligibility criteria

## Key inclusion criteria

1. Patients must have been ≥18 years of age.
2. Patients must have had a confirmed diagnosis of serious bacterial infection, specifically complicated intra-abdominal infection (cIAI), hospital-acquired pneumonia (HAP) or ventilator-associated pneumonia (VAP), complicated urinary tract infection (cUTI) or bloodstream infection (BSI) requiring administration of intravenous (IV) antibacterial therapy.
3. Patients must have had an MBL-positive gram-negative bacteria (an *Enterobacterales* and/or *Stenotrophomonas maltophilia* for which the imipenem or meropenem minimum inhibitory concentrations were ≥4 mg/L) that was isolated from an appropriate specimen obtained within 7 days prior to screening (the study qualifying pathogen, which was determined to be the causative agent of entry infection and which was available to be sent to the central laboratory).
4. Prior to screening, genotypic confirmation of an MBL-positive pathogen at the local laboratory was required. If this was not possible then selected phenotypic tests may have been acceptable with prior approval of the sponsor. In the case of mixed infection, the patient was allowed to participate in the study if the species were deemed susceptible to aztreonam-avibactam or the investigator considered that the additional species were colonizers which did not warrant specific treatment.

### Patients with cIAI

1. Patient must have had a specimen obtained from an abdominal source during a surgical intervention within 7 days prior to screening from which a study-qualifying pathogen was isolated upon culture. Surgical intervention included open laparotomy, percutaneous drainage of an abscess or laparoscopic surgery.

### Patients with HAP/VAP

1. Onset of symptoms >48 hours after admission or <7 days after discharge from an inpatient care facility (for which the duration of admission was >3 days).
2. New or worsening infiltrate on chest X-ray (or computed tomography scan) obtained within 48 hours prior to randomization.
3. At least one of the following:

- Documented fever (temperature ≥38°C) or hypothermia (rectal/core temperature ≤35°C); white blood cells (WBC) ≥10 000 cells/mm^3^, leukopenia with total WBC ≤4500 cells/mm^3^, or >15% immature neutrophils (bands) noted on peripheral blood smear.

### Patients with cUTI

1. Patient had urine within 7 days prior to screening that cultured positive, containing ≥10^5^ colony-forming units (CFU)/mL of at least one carbapenem-nonsusceptible, MBL-positive gram-negative bacteria, i.e. the isolate from the study qualifying culture.
2. Patient had pyuria in the 7 days prior to screening as determined by a midstream clean catch or catheterized urine specimen with ≥10 WBC per high-power field on standard examination of urine sediment or ≥10 WBC/mm^3^ in unspun urine.
3. Patient demonstrated either acute pyelonephritis or complicated lower UTI without pyelonephritis.

### Patients with BSI

1. Patient had a confirmed diagnosis of primary BSI or catheter-related BSI.
2. Signs and symptoms of systemic infection characterized by at least one of the following:

- Chills, rigors or fever (temperature of ≥38.0°C or ≥100.4°F).
- Elevated WBC count (≥10 000/mm^3^) or left shift (>15% immature polymorphonuclear leukocytes).

## Key exclusion criteria

1. Patient had a history of serious allergy such as anaphylaxis, angioedema and bronchospasm, hypersensitivity or any serious reactions to any systemic antibacterial, which is allowed per protocol.
2. Patient had a concurrent infection that may interfere with the evaluation of response to the study antibiotics.
3. Patient had a need for effective concomitant systemic antibacterials in addition to those allowed per protocol for the diagnoses under study.
4. Patient had an estimated creatinine clearance (CrCL) ≤15 mL/min by Cockcroft‑Gault formula, receiving or having had a requirement for peritoneal dialysis, haemodialysis or hemofiltration.
5. Pregnant female patients, breastfeeding female subjects, fertile male patients and female patients of childbearing potential who were unwilling or unable to use a highly effective method of contraception as outlined in this protocol for the duration of the study treatment and for at least 7 days after the last infusion of investigational product.
6. Patient had other acute or chronic medical or psychiatric condition including recent (within the past year) or active suicidal ideation or behaviour or laboratory abnormality that may have increased the risk associated with study participation or investigational product administration or may have interfered with the interpretation of study results and, in the judgment of the investigator, would make the patient inappropriate for entry into this study.

## Study treatments

The recommended treatment duration was 5–14 days for patients with cIAI, cUTI and BSI, and 7–14 days for patients with HAP/VAP.

Patients in the aztreonam-avibactam group received a 500–167 mg aztreonam-avibactam loading dose (30-minute infusion), followed immediately by a 1500–500 mg extended loading dose (3-hour infusions), and then 1500–500 mg maintenance (3-hour infusions) doses every 6 hours. Dosing was adjusted according to renal function for patients with estimated serum CrCL <50 mL/min (see below).

## Aztreonam-avibactam dosage regimens for patients with moderate or severe renal impairment

| **Creatinine clearance, mL/min^a^** | **LD, aztreonam / avibactam (30-minute IV infusion)** | **ELD, aztreonam / avibactam (3-hour IV infusion)** | **Time between end of ELD and first MD** | **MD, aztreonam / avibactam (3-hour IV infusion)** | **Frequency of MD** |
| --- | --- | --- | --- | --- | --- |
| >50 | 500 mg / 167 mg | 1500 mg / 500 mg | 3 hours | 1500 mg / 500 mg | q6h |
| >30 to 50 | 500 mg / 167 mg | 1500 mg / 500 mg | 3 hours | 750 mg / 250 mg | q6h |
| >15 to 30 | 675 mg / 225 mg | 675 mg / 225 mg | 5 hours | 675 mg / 225 mg | q8h |

^a^Estimated using the Cockcroft-Gualt formula.
ELD, extended loading dose; IV, intravenous; LD, loading dose; MD, maintenance dose; q6h, every 6 hours;
q8h, every 8 hours.

While the aztreonam-avibactam dosing regimen in the Phase 3 trial programme included two loading doses administered sequentially followed by regular maintenance doses, the approved European doses include a simplified dosing regimen with a single loading dose.

Patients in the BAT group could receive monotherapy or combination therapy at the investigators’ discretion, in accordance with study site standard practice and local epidemiology.

In the aztreonam-avibactam group, patients with cIAI also received metronidazole 500 mg IV every 8 hours (q8h; by 60-minute IV infusion). Patients with cIAI in the BAT group could receive metronidazole 500 mg IV q8h (by 60-minute IV infusion) if BAT was not deemed to provide adequate anaerobic coverage.

All patients could also receive optional gram-positive coverage at the investigators’ discretion, and those with HAP/VAP and proven or suspected co-infection with *Pseudomonas aeruginosa* could also receive optional IV aminoglycoside.

## Description of analysis sets

| **Analysis set** | **Description** |
| --- | --- |
| ITT | The ITT analysis set included all randomized patients regardless of receipt of study drug. Patients in the ITT analysis set were analysed according to the treatment to which they were randomized. |
| Micro-ITT | This subset of the ITT analysis set included all patients who had at least one MBL‑positive, gram-negative baseline pathogen from an adequate specimen obtained prior to the start of study treatment.  Patients with inherently resistant pathogens (for example, monomicrobial infections due to any *Acinetobacter* spp.) were excluded from the micro-ITT analysis set. |
| ME | This subset of the micro-ITT analysis set included all patients who met the definition of the micro-ITT analysis set AND   - - received ≥48 hours of study drug OR received <48 hours of study drug before discontinuing study drug due to an AE;   - did not receive concomitant antibiotic therapy with potential activity against any baseline MBL-positive pathogens between the time of the first dose of study treatment and the time of TOC. This did not include those patients whose study therapy had failed and required additional antibiotics to treat their infection;   - had the baseline entry organism(s) genetically confirmed by central microbiological testing;   - did not have a clinical outcome of indeterminate at TOC. |
| Safety | This subset of the ITT analysis set included all patients who received any amount of study treatment.  Patients in the safety analysis set were analysed according to the treatment that they received. |

AE, adverse event; MBL, metallo-β-lactamase; ME, microbiologically evaluable; micro-ITT, microbiological ITT; TOC, test-of-cure.

## Efficacy assessments

Clinical response and microbiological responses were assessed by investigators at the EOT and TOC visits. Clinical responses were categorized as either cure, failure or indeterminate (see below).

**Definition of clinical response categories at the EOT and TOC visits**

| **Response** | **Definition** |
| --- | --- |
| Clinical cure | Baseline signs and symptoms had improved such that after study treatment, no further antimicrobial treatment for the index infection (i.e. cIAI, cUTI, HAP/VAP or BSI) was required.^a^  In addition, none of the clinical failure criteria listed below were met.  Additionally, for cIAI patients: No unplanned drainage or surgical intervention was necessary since the initial procedure. |
| Clinical failure | Patients’ clinical responses that met any of the following criteria were considered a treatment failure:   - Death (after receiving ≥48 hours of study treatment). - Received treatment with further antibiotics for the index infection.^a^ This includes subjects prematurely discontinued from study treatment due to an AE who required further antibiotics for the index infection. - Additionally, for patients with cIAI: persisting or recurrent infection within the abdomen documented by the findings at re-intervention either percutaneously or operatively in situation of adequate infection source control at the time of initial surgical procedure. Postsurgical wound infections (e.g. signs of local infection such as purulent exudates, erythema or warmth that requires additional antibiotics and/or non-routine wound care). |
| Indeterminate | Death (after receiving <48 hours of study treatment).  Patient lost to follow-up such that a determination of clinical response cannot be made.  Additionally, for cIAI patients: Inadequate infection source control at time of initial surgical procedure. |

^a^Further antibiotics for the index infection should only be initiated for ongoing or worsening signs and symptoms of the infection.

AE, adverse event; BSI, bloodstream infection; cIAI, complicated intra-abdominal infection; cUTI, complicated urinary tract infection; EOT, end-of-treatment; HAP, hospital-acquired pneumonia; VAP, ventilator-associated pneumonia; TOC, test-of-cure.

Clinical response outcomes were subsequently assessed by an independent adjudication committee blinded to treatment assignment with the aim of unbiased adjudication of the primary objective measure. Data were provided relating to the subject’s clinical response (e.g. death status, disease progression, adverse events (AEs), surgical procedures) without disclosing treatment arm. In case of a discrepancy with the investigator’s assignment of clinical response, the adjudication committee’s assessment would prevail for the primary analysis.

Per-patient and per-pathogen microbiological responses at EOT and TOC were classified as favourable, unfavourable or indeterminate (see below).

## Definition of microbiological response categories at the EOT and TOC visits

| Response | Definition |
| --- | --- |
| **Favourable** |  |
| Eradication | Absence of causative pathogen from an appropriately obtained specimen^a^ at the site of infection. |
| Presumed eradication | Repeat culture of specimens were not performed/clinically indicated in a patient who had a clinical response of cure. |
| **Unfavourable** |  |
| Persistence | Causative organism is still present from an appropriately obtained specimen at the site of infection. If the causative organism displays ≥4-fold higher mic to study therapy after treatment with IV study therapy, the response will also be categorized as persistence with increasing MIC. |
| Presumed persistence | Patient was assessed as a clinical failure and repeat culture of specimens were not performed/clinically indicated. |
| **Indeterminate** | - Death (after receiving less than 48 hours of study treatment). - Patient lost to follow-up such that a determination of microbiological response cannot be made. - Additionally, for cIAI patients: inadequate infection source control at time of initial surgical procedure. |

^a^For patients with cIAI, an appropriately obtained specimen for determination of microbiological response was defined as a specimen obtained using an adequate technique (e.g., surgical procedure [laparotomy or laparoscopy], percutaneous drainage [where in place for <24 hours], or wounds where the patient had a superficial or deep surgical wound reported at any point during the follow-up period). From expectorated or induced sputum, an adequate specimen is one with ≤10 squamous epithelial cells and >25 polymorphonuclear neutrophils per low power field upon a gram-stain; throat secretions are considered to be inadequate; other specimens such as endotracheal aspirate, BAL, mini-BAL and PSB were considered to be adequate. For blood, two sets of blood cultures were to be collected (i.e. four bottles) from two different sites for aerobic and anaerobic incubation. One set of blood cultures must have been obtained through a venipuncture.

BAL, bronchoalveolar lavage; cIAI, complicated intra-abdominal infection; EOT, end of treatment; IV, intravenous; MIC, minimum inhibitory concentration; PSB, protected specimen brush: TOC, test-of-cure.

## Safety assessments

Safety assessments included treatment-emergent AEs (monitored throughout the study), vital signs, physical examinations, laboratory assessments, liver function tests and electrocardiograms. An external data monitoring committee performed ongoing monitoring of the safety of patients during the study via interim safety reviews.

## Statistical methods

The study planned to randomize approximately 60 patients in a 2:1 ratio. As no formal hypothesis testing was planned for this study, no power calculation was carried out to assess the number of patients required for each treatment arm. The primary efficacy analysis was the estimated clinical cure rate at TOC in the micro-ITT analysis set. Single-arm 95% confidence intervals for the estimated clinical cure rate in each treatment group were computed using Jeffrey’s method.

AEs were coded by system organ class and preferred term using Medical Dictionary for Regulatory Activities (MedDRA) v25.1. Predefined standardized MedDRA queries (narrow and broad scope) were used to evaluate AEs of special interest (liver disorders, *Clostridioides difficile*-associated diarrhoea and hypersensitivity/anaphylaxis).

# Supplementary results

## *Patients*

Of 15 patients randomized (aztreonam-avibactam, *n* = 12; BAT, *n* = 3), two patients with cIAI, three with HAP/VAP, three with cUTI and four with BSI received aztreonam-avibactam; and one patient each with HAP/VAP, cUTI and BSI were assigned to BAT (Figure S1).

**Figure S1. Trial profile**


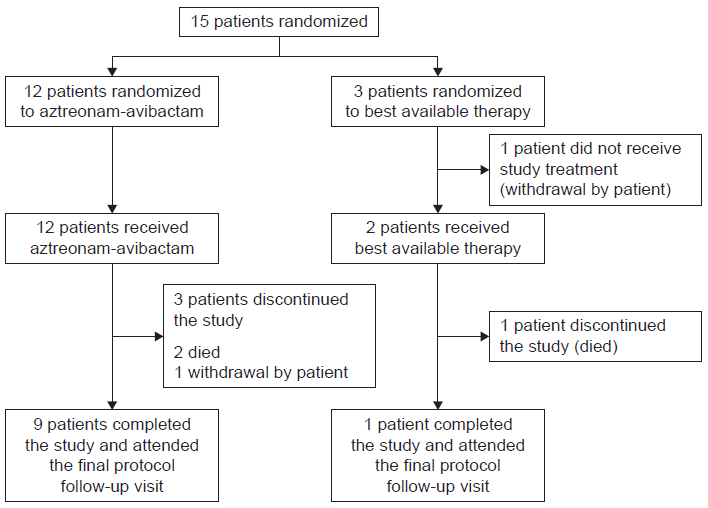


The mean age of patients was 58.4 years and 60% of patients were male. Most patients were White or Asian. Eight of the 12 patients randomized to aztreonam-avibactam and two of the three patients randomized to BAT had previous treatment failure (Table 1).

The median (range) duration of treatment (safety analysis set) was 9.5 (1–15) days for aztreonam-avibactam and 4.5 (4–5) days for BAT. For the two patients who received BAT, treatment consisted of amikacin + polymyxin + meropenem (*n* = 1) and amikacin + colistin (*n* = 1). In the aztreonam-avibactam group, one patient received gram-positive antibiotic treatment (vancomycin) during the study treatment period. Excluding patients who received an aminoglycoside as part of BAT, no patients received optional aminoglycosides during the study treatment period.

## *Baseline pathogens*

*Escherichia coli* was identified in two (17%) patients in the aztreonam-avibactam group and in one (33%) patient in the BAT group. Other baseline pathogens identified in the aztreonam-avibactam group included *S. maltophilia* (*n* = 3 [25%]), *P. aeruginosa* (*n* = 2 [17%]), *K. pneumoniae* complex (*n* = 2 [17%]), *Enterobacter cloacae* complex (*n* = 1 [8%]), *Klebsiella aerogenes* (*n* = 1 [8%]) and *Enterococcus faecium* (*n* = 1 [8%]).

Six patients in the aztreonam-avibactam group (none in the BAT group) had polymicrobial infections, three with two MBL-positive pathogens each, two with one MBL-positive and one serine carbapenemase-positive pathogen each, and one with an MBL-positive pathogen and a gram-positive pathogen (*E. faecium*). Baseline resistance characteristics are shown in Table S1.

**Table S1. Baseline pathogen resistance characteristics (micro-ITT analysis set)**

|  | **Aztreonam-avibactam (*n* = 12)** | **Best available therapy (*n* = 3)** |
| --- | --- | --- |
| **Number of baseline pathogens** | 18 | 3 |
| Number of baseline aerobic, gram-negative pathogens | 17 | 3 |
| Number of baseline aerobic, gram-positive pathogens | 1 | 0 |
| Number of baseline Enterobacterales pathogens | 12 | 3 |
| Number of baseline aerobic, gram-negative, non-Enterobacterales pathogens | 5 | 0 |
| **Aztreonam or meropenem susceptibility^a^** |  |  |
| Number of isolates tested | 14 | 3 |
| Aztreonam non-susceptible (CLSI) | 11 (78.6) | 3 (100) |
| Aztreonam non-susceptible (EUCAST) | 13 (92.9) | 3 (100) |
| Meropenem non-susceptible (CLSI) | 14 (100) | 3 (100) |
| Meropenem non-susceptible (EUCAST) | 14 (100) | 3 (100) |
| **ESBLs^b^** |  |  |
| Number of isolates tested | 12 | 3 |
| ESBL-positive | 10 (83.3) | 3 (100) |
| **Carbapenemases^c^** |  |  |
| Number of isolates tested | 17 | 3 |
| Carbapenemase-positive | 17 (100) | 3 (100) |
| Serine carbapenemase-positive^d^ | 3 (17.6) | 0 |
| OXA-48 | 2 (11.7) | 0 |
| OXA-232 | 1 (5.9) | 0 |
| MBL-positive^d^ | 15 (88.2) | 3 (100) |
| VIM | 2 (13.3) | 0 |
| NDM | 10 (66.7) | 3 (100) |
| L1 | 3 (20.0) | 0 |

^a^Aztreonam or meropenem susceptibility testing was done for all baseline aerobic gram-negative pathogens excluding *Stenotrophomonas maltophilia*. Percentages are based on the number of baseline aerobic gram‑negative pathogens excluding *S. maltophilia*.

^b^ESBL testing was done for all baseline Enterobacterales pathogens. Percentages are based on the number of baseline Enterobacterales pathogens.

^c^Carbapenemase testing was done for Enterobacterales and *Pseudomonas aeruginosa* that were resistant to aztreonam, ceftazidime, meropenem, or imipenem by MIC testing, or that had aztreonam-avibactam MIC ≥4 mg/L. *S. maltophilia* was not tested for carbapenemase but was included in carbapenemase-positive counts as it is inherently carbapenemase-positive due to the presence of the L1 MBL subtype. Percentages are based on the number of baseline aerobic gram-negative pathogens.

^d^One isolate in one patient was serine carbapenemase­-positive (OXA-48) and MBL-positive (NDM).

CLSI, Clinical and Laboratory Standards Institute; ESBL, extended-spectrum β‑lactamases; EUCAST, European Committee on Antimicrobial Susceptibility Testing; MBL, metallo-β-lactamases; MIC, minimum inhibitory concentration; micro-ITT, microbiological ITT; NDM, New Delhi MBL; VIM, Verona integron-encoded MBL.

MBL subtypes and variants identified in the aztreonam-avibactam group were: NDM-1 (*n* = 7), NDM-5 (*n* = 3), VIM-2 (*n* = 2) and L1 (*n* = 3); and in the BAT group were NDM-1 (*n* = 2) and NDM-5 (*n* = 1) (Table 1 and Table S2).

Table S2. Patient-level summary of baseline pathogen characteristics, treatment duration, clinical outcomes, SAEs and narratives (micro-ITT analysis set)

| Patient  Sex / age (years)  Indication | Treatment / duration | Prior Abx failure | Renal function | Baseline pathogens  MICs | MBLs  Other β-lactamase enzyme(s)^a^ | Adjudicated response at TOC | | Survival (28-day) | SAEs | Additional information |
| --- | --- | --- | --- | --- | --- | --- | --- | --- | --- | --- |
|  |  |  |  |  |  | Clinical | Microbiological |  |  |  |
| Male / 83  cUTI | ATM-AVI / 7 days | No | Severe impairment | *Klebsiella pneumoniae* complex  ATM MIC >64 µg/mL  ATM-AVI MIC 0.12 µg/mL | **NDM-1**  CTX-M-15, OXA-1, SHV-28, TEM-1 | Cure | Favourable | Alive | Yes | The patient experienced an SAE of urinary tract obstruction on study day 3 that resolved on study day 5. Both the investigator and the clinical adjudication committee assessed the patient as a clinical cure at TOC. |
| Female / 31  VAP (polymicrobial) | ATM-AVI / 14 days | No | Augmented | *Pseudomonas aeruginosa*  ATM MIC >8 µg/mL  ATM-AVI MIC 8 µg/mL | **VIM-2**  OXA-488, PDC-35, PA5542-like | Failure | Unfavourable | Alive | No | The patient experienced an AE of large intestinal anastomotic leak on study day 1, which was treated with metronidazole, and an AE of bacteraemia (*Enterococcus faecalis* from blood culture) on study day 9 during the study treatment phase. Though the AEs on study days 1 and 9 may not have been related to the index infection of VAP, both the investigator and the clinical adjudication committee assessed the patient as having had treatment failure at TOC. |
|  |  |  |  | *K. pneumoniae*  ATM MIC >64 µg/mL  ATM-AVI MIC 0.25 µg/mL | CTX-M-15, OXA-1, OXA-48, SHV-11, TEM-1 |  |  |  |  |  |
| Male / 60  cIAI (polymicrobial) | ATM-AVI / 5 days | Yes | Normal/mild impairment | *Escherichia coli*  ATM MIC >64 µg/mL  ATM-AVI MIC 8 µg/mL | **NDM-5**  CMY-145, EC-11-like, TEM-1 | Failure | Unfavourable | Alive | No | On study day 2, oral erythromycin was administered as a prokinetic agent for medical history of ileus. The participant response was considered a cure by the investigator at the EOT visit. Clinical response was assessed as a failure by the clinical adjudication committee at EOT (and TOC). The patient withdrew from the study for personal reasons on study day 28 and therefore never completed the TOC visit. No AEs were reported for this subject. |
|  |  |  |  | *K. pneumoniae*  ATM MIC 4 µg/mL  ATM-AVI MIC 0.5 µg/mL | OXA-1, OXA-232, SHV-11, SHV-31 |  |  |  |  |  |
| Male / 32  cUTI (polymicrobial) | ATM-AVI / 14 days | Yes | Normal/mild impairment | *K. pneumoniae*  ATM MIC >64 µg/mL  ATM-AVI MIC 0.25 µg/mL | **NDM-1**  CTX-M-15, OXA-1, SHV-11, TEM-1 | Cure | Favourable | Alive | No | Both the investigator and the clinical adjudication committee assessed the patient response as a clinical cure at TOC. |
|  |  |  |  | *K. pneumoniae* complex  ATM MIC >64 µg/mL  ATM-AVI MIC 0.25 µg/mL | **NDM-1**  CTX-M-15, OXA-1, SHV-11, TEM-1 |  |  |  |  |  |
| Female / 38  cIAI (polymicrobial) | ATM-AVI / 3 days | Yes | Normal/mild impairment | *Klebsiella aerogenes*  ATM MIC 0.12 µg/mL  ATM-AVI MIC 0.12 µg/mL | **NDM-5**  *amp*C-like, LAP-2 | Failure | Unfavourable | Alive | No | Prior antibiotic therapy was given for 17 days prior to randomization. The patient received ATM-AVI and metronidazole for 3 days during the treatment phase (and VAN for 32 days), at which time the investigator considered that the clinical response was failure at the EOT visit (as did the clinical adjudication committee). Both the investigator and the clinical adjudication committee assessed the participant response as a failure at TOC. |
|  |  |  |  | *Enterococcus faecium*  VAN MIC 0.5 µg/mL | N/A |  |  |  |  |  |
| Male / 51  BSI | ATM-AVI / 12 days | Yes | Normal/mild impairment | *Stenotrophomonas maltophilia*  ATM MIC >64 µg/mL  ATM-AVI MIC 2 µg/mL | **L1** | Cure | Favourable | Alive | No | Both the investigator and the clinical adjudication committee assessed the patient response as a clinical cure at TOC. |
| Male / 62  HAP | ATM-AVI / 10 days | Yes | Augmented | *P. aeruginosa*  ATM MIC 32 µg/mL  ATM-AVI MIC 16 µg/mL | **VIM-2**  OXA-10, OXA-488, PDC-35, PA5542-like | Indeterminate | Indeterminate | Alive | No | The investigator considered the patient response to be a clinical cure at the EOT visit (as did the clinical adjudication committee) but could not perform a TOC visit because the patient did not attend the study visit (per protocol, lost to follow-up is a criterion for an indeterminate outcome). |
| Male / 45  VAP (polymicrobial) | ATM-AVI / 1 day | Yes | Augmented | *K. pneumoniae*  ATM MIC >64 µg/mL  ATM-AVI MIC 0.5 µg/mL | **NDM-1**  CTX-M-15, OXA-9, OXA-48, SHV-1, SHV-12, TEM-1 | Indeterminate | Indeterminate | Death (day 1) | Yes | The patient died on study day 1 due to multiple organ dysfunction syndrome and sepsis. The participant only received one dose of ATM-AVI (loading dose and extended loading dose), and therefore was adjudicated as an indeterminate clinical outcome because death occurred after receiving less than 48 hours of study treatment (per protocol, less than 48 hours of treatment is an indeterminate outcome. |
|  |  |  |  | *S. maltophilia*^c^ | **L1** |  |  |  |  |  |
| Female / 62  BSI | ATM-AVI / 14 days | No | Moderate impairment | *K. pneumoniae*  ATM MIC >64 µg/mL  ATM-AVI MIC 0.12 µg/mL | **NDM-1**  CTX-M-15, SHV-11, TEM-1 | Cure | Favourable | Alive | Yes | The patient had an SAE of acute pulmonary oedema on study day 5. Both the investigator and the clinical adjudication committee assessed the patient response as a clinical cure at TOC. |
| Male / 73  BSI (polymicrobial) | ATM-AVI / 14 days | Yes | Normal/mild impairment | *E. coli*  ATM MIC >64 µg/mL  ATM-AVI MIC 1 µg/mL | **NDM-5**  CMY-2, CTX-M-15, EC-11-like, OXA-1, TEM-1 | Failure | Unfavourable | Alive | Yes | Patient experienced an AE of *E. faecalis* isolated from a blood culture on study day 6, for which ampicillin was given. At the EOT visit, the investigator considered the patient response to be a cure (assessed as failure by the clinical adjudication committee).  Patient experienced AEs of thrombophlebitis and *Ochrobacterium intermedium* isolated from a blood culture on study day 12 (at the EOT visit), for which ciprofloxacin was given.  A TOC visit was not performed. The patient died on day 31 from neoplasm progression. |
|  |  |  |  | *S. maltophilia*  ATM MIC >64 µg/mL  ATM-AVI MIC 8 µg/mL | **L1** |  |  |  |  |  |
| Male / 73  cUTI | ATM-AVI / 5 days | Yes | Normal/mild impairment | *Enterobacter cloacae* complex  ATM MIC >64 µg/mL  ATM-AVI MIC 1 µg/mL | **NDM-1**  ACT-24, CTX-M-15, OXA-1, TEM-1 | Failure | Favourable | Alive | Yes | Both the investigator and the clinical adjudication committee assessed the patient response as a cure at EOT. The patient experienced an AE of complicated pyelonephritis on study day 14, for which meropenem was given. A urine culture was positive for *E. cloacae.* The patient response was considered a cure by the investigator but assessed as a failure by the clinical adjudication committee at TOC. |
| Female / 69  BSI | ATM-AVI / 5 days | No | Normal/mild impairment | *K. pneumoniae*  ATM MIC >64 µg/mL  ATM-AVI MIC 0.5 µg/mL | **NDM-1**  CTX-M-15, OXA-9, SHV-1, SHV-12, TEM-1 | Cure | Favourable | Alive | No | Both the investigator and the clinical adjudication committee assessed the patient response as a clinical cure at TOC. |
| Female / 73  cUTI | BAT / 0 days | Yes | Normal | *E. coli*  ATM MIC >64 µg/mL  ATM-AVI MIC 1 µg/mL | **NDM-5**  CTX-M-15, EC-5-like, TEM-1-like | Indeterminate | Indeterminate | Unknown^d^ | N/A^d^ | Patient was randomized to BAT but did not receive study therapy as patient withdrew from study participation prior to first dose. Since study treatment was never received, this case was not presented to the clinical adjudication committee; the clinical response was derived as indeterminate. |
| Male / 60  BSI | AMK / 5 days  COL / 5 days | No | Augmented | *K. pneumoniae*  ATM MIC >64 µg/mL  ATM-AVI MIC 0.25 µg/mL  AMK MIC 32 µg/mL  COL MIC >32 µg/mL | **NDM-1**  CTX-M-15, OXA-1, SHV-11, TEM-1 | Failure | Unfavourable | Alive | Yes | Patient discontinued from study treatment at Day 5 due to a treatment-related SAE of acute kidney injury and therefore required further antibiotics for the index infection. Both the investigator and the clinical adjudication committee assessed the patient response as a failure at TOC. |
| Female / 64  VAP | MER / 3 days  AMK / 2 days  Polymixin / 2 days | Yes | Unknown | *K. pneumoniae*  AMK MIC 16 µg/mL  MER MIC >8 µg/mL  ATM MIC >64 µg/mL  ATM-AVI MIC 0.03 µg/mL | **NDM-1**  CTX-M-3, CTX-M-15, OXA-9, SHV-28, TEM-1 | Indeterminate | Indeterminate | Death (day 3) | Yes | The patient died from cardiac arrest and multiple organ dysfunction syndrome on study day 3. The patient was adjudicated as an indeterminate clinical outcome because death occurred after receiving less than 48 hours of study treatment. |

^a^MBLs are in shown in **bold** and serine carbapenemases in red. The beta-lactam enzyme designations were derived from whole genome sequencing of the isolates.

^b^Reported as persistence with increasing MIC.

^c^Isolate was obtained at local laboratory but was not sent to the central laboratory; susceptibility data not available for this isolate.

^d^Patient consent withdrawn before treatment.

Abx, antibiotic; AE, adverse event; AMK, amikacin; ATM, aztreonam; AVI, avibactam; BAT, best available therapy; BSI, bloodstream infection; cIAI, complicated
intra-abdominal infection; COL, colistin; cUTI, complicated urinary tract infection; EOT, end-of-treatment; HAP, hospital-acquired pneumonia; MBL, metallo-β‑lactamase;
MER, meropenem; MIC, minimum inhibitory concentration; micro-ITT, microbiological ITT; N/A, not applicable; NDM, New Delhi MBL; SAE, serious AE; TOC, test-of-cure; VAN, vancomycin; VAP, ventilator-acquired pneumonia; VIM, Verona integron-encoded MBL.

## *Efficacy*

Adjudicated clinical cure rates at TOC in the ME analysis set, which excluded patients who received <48 hours of study treatment, those with an indeterminate response at TOC, and those with ineligible baseline pathogens, were consistent with the primary analysis (Table S3).

**Table S3. Adjudicated clinical responses at TOC (ME analysis set)**

|  | ***n* (%)** | **Aztreonam-avibactam (*n* = 9)** | **Best available therapy (*n* = 1)** |
| --- | --- | --- | --- |
| All patients | *n* | 9 | 1 |
|  | Cure | 5 (55.6) | 0 |
|  | Failure | 4 (44.4) | 1 (33.3) |
| cIAI | *n* | 2 | 0 |
|  | Cure | 0 | – |
|  | Failure | 2 (100.0) | – |
| HAP/VAP | *n* | 0 | 0 |
|  | Cure | – | – |
|  | Failure | – | – |
| cUTI | *n* | 3 | 0 |
|  | Cure | 2 (66.7) | – |
|  | Failure | 1 (33.3) | – |
| BSI | *n* | 4 | 1 |
|  | Cure | 3 (75.0) | 0 |
|  | Failure | 1 (25.0) | (100.0) |

Clinical cure was defined as improvement in baseline signs and symptoms such that no further antimicrobial treatment for the index infection was required, and for patients with cIAI, no unplanned drainage or surgical intervention was necessary since the initial procedure.
Percentages are based on the total number of patients in the treatment arm (*n*).

BSI, bloodstream infection; CI, confidence interval; cIAI, complicated intra-abdominal infection; cUTI, complicated urinary tract infection; HAP, hospital-acquired pneumonia; ME, microbiologically evaluable; TOC, test-of-cure; VAP, ventilator-associated pneumonia.

Of nine patients in the aztreonam-avibactam group with NDM-positive baseline isolates, four had clinical cure at TOC, four had failure and one was indeterminate. Of the four patients with adjudicated clinical failure, three were considered a clinical cure at EOT and/or TOC by the investigator, even though considered a failure at TOC by the Adjudication committee, and all four patients also had prior treatment failure in response to other antibiotics (Table S2). Of two patients with VIM-positive isolates, one had failure and one was indeterminate. Of three patients with L1-positive isolates, one had clinical cure, one had failure and one was indeterminate (Table S2). The patient with adjudicated clinical failure was considered a cure at EOT by the investigator, even though they were considered a failure at EOT by the Adjudication committee (this patient died from neoplasm progression prior to the TOC visit being carried out [Table S2]).

Of the three patients in the BAT group, all of whom had NDM-positive baseline isolates, one was a clinical failure at TOC; this patient was found to have pathogens non-susceptible to the amikacin (MIC 32 mg/L) and colistin (MIC >32 mg/L) BAT administered. The other two patients had indeterminate responses (one of these patients was randomized but did not receive treatment and one died after having received <48 hours of study treatment; death was due to cardiac arrest and multiple organ dysfunction) (Table S2).

In the aztreonam-avibactam group, two of the five patients with clinical cure at TOC had previous treatment failure. Both patients with an indeterminate clinical response had previous treatment failure (one had a clinical cure at the end-of-treatment (EOT) visit but lost to follow-up at TOC; one died after receiving a single dose of aztreonam-avibactam, due to multiple organ dysfunction syndrome and sepsis considered unrelated to the study drug). Four of the five patients with clinical failure at TOC had previous treatment failure. In the BAT group, the patient with clinical failure at TOC did not have a previous treatment failure; both patients with an indeterminate clinical response had previous treatment failure, of whom one did not receive study treatment and the other died on study day 3 (Table S2).

Table S4. Per-pathogen microbiological responses at TOC (micro-ITT analysis set)

| Baseline pathogen | Response, *n* (%) | Aztreonam-avibactam (*n* = 12) | Best available therapy (*n* = 3) |
| --- | --- | --- | --- |
| Enterobacterales |  |  |  |
| All Enterobacterales | n | 12 | 3 |
|  | Favourable | 6 (50.0) | 0 |
|  | Eradication | 5 (41.7) | 0 |
|  | Presumed eradication | 1 (8.3) | 0 |
|  | Unfavourable | 5 (41.7) | 1 (33.3) |
|  | Presumed persistence | 5 (41.7) | 1 (33.3) |
|  | Indeterminate | 1 (8.3) | 2 (66.7) |
| Other gram-negative aerobes |  |  |  |
| *Pseudomonas aeruginosa* | n | 2 | 0 |
|  | Favourable | 0 | – |
|  | Unfavourable | 1 (50.0) | – |
|  | Persistence | 1 (50.0) | – |
|  | Persistence with increasing MIC^a^ | 1 (50.0) | – |
|  | Indeterminate | 1 (50.0) | – |
| *Stenotrophomonas maltophilia* | n | 3 | 0 |
|  | Favourable | 1 (33.3) | – |
|  | Presumed eradication | 1 (33.3) | – |
|  | Unfavourable | 1 (33.3) | – |
|  | Presumed persistence | 1 (33.3) | – |
|  | Indeterminate | 1 (33.3) | – |
| Gram-positive aerobes |  |  |  |
| *Enterococcus faecium* | n | 1 | 0 |
|  | Favourable | 0 | – |
|  | Unfavourable | 1 (100) | – |
|  | Presumed persistence | 1 (100) | – |

Patients could have ≥1 pathogen. Multiple isolates of the same species from the same patient are counted only once, regardless of the source (blood, respiratory, or sputum).

^a^Persistence with increasing MIC defined as ≥4-fold increase in the MIC for the study drug received compared to the baseline isolate.

MIC, minimum inhibitory concentration; micro-ITT; microbiological ITT; TOC, test-of-cure.

Table S5. Proportion of patients who died within 28 days of randomization (ITT analysis set)

|  |  | **Aztreonam-avibactam (*n* = 12)** | **Best available therapy (*n* = 3)** |
| --- | --- | --- | --- |
| All participants | *n* | 12 | 3 |
|  | Death within 28 days after randomization, *n* (%) | 1 (8.3) | 1 (33.3) |
|  | Disease under study, *n* (%) | 1 (8.3) | 0 |
|  | Other | 0 | 1 (33.3)^a^ |
| cIAI | *n* | 2 | 0 |
|  | Death within 28 days after randomization | 0 | 0 |
| HAP/VAP | *n* | 3 | 1 |
|  | Death within 28 days after randomization, *n* (%) | 1 (33.3) | 1 (100.0) |
|  | Disease under study, *n* (%) | 1 (33.3) | 0 |
|  | Other | 0 | 1 (100.0) |
| cUTI | *n* | 3 | 1 |
|  | Death within 28 days after randomization | 0 | 0 |
| BSI | *n* | 4 | 1 |
|  | Death within 28 days after randomization | 0 | 0 |
|  |  |  |  |

^a^Death due to neoplasm progression.

BSI, bloodstream infection; cIAI, complicated intra-abdominal infection; cUTI, complicated urinary tract infection; HAP, hospital-acquired pneumonia; VAP, ventilator-associated pneumonia.

## *Safety*

## Table S6. Adverse events (safety analysis set)

| *n* (%) | Aztreonam-avibactam (*n* = 12) | Best available therapy (*n* = 2) |
| --- | --- | --- |
| Number of AEs | 35 | 5 |
| Patients with at least one AE | 11 (91.7) | 2 (100.0) |
| Patients with at least one treatment-related AE | 2 (16.7) | 1 (50.0) |
| Patients with at least one SAE | 5 (41.7) | 2 (100.0) |
| Patients with at least one severe AE | 4 (33.3) | 1 (50.0) |
| Patients with fatal AEs | 2 (16.7) | 1 (50.0) |
| Patients discontinued from study due to AEs | 2 (16.7) | 1 (50.0) |
| Patients discontinued study drug due to AE | 1 (8.3) | 2 (100.0) |

AE, adverse event; SAE, serious AE.

Table S7. Overview of all-cause AEs (safety analysis set)

| **Patients with AEs by system organ class**  **and preferred term, *n* (%)** | **Aztreonam-avibactam (*n* = 12)** | **Best available therapy (*n* = 2)** |  |
| --- | --- | --- | --- |
| **Patients with at least one AE** | **11 (91.7)** | **2 (100.0)** |  |
| Blood and lymphatic system disorders | 1 (8.3) | 0 |  |
| Thrombocytopenia | 1 (8.3) | 0 |  |
| Cardiac disorders | 0 | 1 (50.0) |  |
| Cardiac arrest | 0 | 1 (50.0) |  |
| Eye disorders | 1 (8.3) | 0 |  |
| Ocular hypertension | 1 (8.3) | 0 |  |
| Gastrointestinal disorders | 1 (8.3) | 0 |  |
| Frequent bowel movements | 1 (8.3) | 0 |  |
| General disorders and administration site conditions | 2 (16.7) | 2 (100.0) | |
| Chest discomfort | 1 (8.3) | 0 |  |
| Multiple organ dysfunction syndrome | 1 (8.3) | 1 (50.0) |  |
| Pyrexia | 0 | 1 (50.0) |  |
| Hepatobiliary disorders | 1 (8.3) | 0 |  |
| Hepatic function abnormal | 1 (8.3) | 0 |  |
| Infections and infestations | 4 (33.3) | 1 (50.0) |  |
| Bacteraemia | 1 (8.3) | 0 |  |
| Intervertebral discitis | 1 (8.3) | 0 |  |
| Pyelonephritis | 1 (8.3) | 0 |  |
| Sepsis | 1 (8.3) | 0 |  |
| Urinary tract infection | 0 | 1 (50.0) |  |
| Injury, poisoning and procedural complications | 2 (16.7) | 0 |  |
| Abdominal wound dehiscence | 1 (8.3) | 0 |  |
| Stoma prolapse | 1 (8.3) | 0 |  |
| Investigations | 3 (25.0) | 0 |  |
| Alanine aminotransferase increased | 1 (8.3) | 0 |  |
| Aspartate aminotransferase increased | 1 (8.3) | 0 |  |
| Bacterial test positive | 1 (8.3) | 0 |  |
| Blood alkaline phosphatase increased | 1 (8.3) | 0 |  |
| Blood creatinine increased | 1 (8.3) | 0 |  |
| Catheter culture positive | 1 (8.3) | 0 |  |
| Enterococcus test positive | 1 (8.3) | 0 |  |
| Gamma-glutamyl transferase increased | 1 (8.3) | 0 |  |
| Platelet count increased | 1 (8.3) | 0 |  |
| Staphylococcus test positive | 1 (8.3) | 0 |  |
| Metabolism and nutrition disorders | 2 (16.7) | 0 |  |
| Hypokalaemia | 2 (16.7) | 0 |  |
| Neoplasms benign, malignant and unspecified (including cysts and polyps) | 1 (8.3) | 0 |  |
| Neoplasm progression | 1 (8.3) | 0 |  |
| Product issues | 1 (8.3) | 0 |  |
| Device occlusion | 1 (8.3) | 0 |  |
| Psychiatric disorders | 1 (8.3) | 0 |  |
| Intensive care unit delirium | 1 (8.3) | 0 |  |
| Renal and urinary disorders | 2 (16.7) | 1 (50.0) |  |
| Acute kidney injury | 0 | 1 (50.0) |  |
| Ureterolithiasis | 1 (8.3) | 0 |  |
| Urinary tract obstruction | 1 (8.3) | 0 |  |
| Respiratory, thoracic and mediastinal disorders | 1 (8.3) | 0 |  |
| Acute pulmonary oedema | 1 (8.3) | 0 |  |
| Skin and subcutaneous tissue disorders | 2 (16.7) | 0 |  |
| Decubitus ulcer | 1 (8.3) | 0 |  |
| Skin maceration | 1 (8.3) | 0 |  |
| Vascular disorders | 2 (16.7) | 0 |  |
| Hematoma | 1 (8.3) | 0 |  |
| Phlebitis | 1 (8.3) | 0 |  |
| Thrombophlebitis | 1 (8.3) | 0 |  |

AEs were coded by system organ class and preferred term using MedDRA v25.1.

AE, adverse event; MedDRA, Medical Dictionary for Regulatory Activities.

Table S8. Overview of all-cause SAEs (safety analysis set)

| **Patients with SAEs by system organ class**  **and preferred term, *n* (%)** | **Aztreonam-avibactam (*n* = 12)** | **Best available therapy (*n* = 2)** |
| --- | --- | --- |
| Patients with at least one SAE | 5 (41.7) | 2 (100.0) |
| Cardiac disorders | 0 | 1 (50.0) |
| Cardiac arrest | 0 | 1 (50.0) |
| General disorders and administration site conditions | 1 (8.3) | 1 (50.0) |
| Multiple organ dysfunction syndrome | 1 (8.3) | 1 (50.0) |
| Infections and infestations | 2 (16.7) | 1 (50.0) |
| Pyelonephritis | 1 (8.3) | 0 |
| Sepsis | 1 (8.3) | 0 |
| Urinary tract infection | 0 | 1 (50.0) |
| Investigations | 1 (8.3) | 0 |
| Bacterial test positive | 1 (8.3) | 0 |
| Enterococcus test positive | 1 (8.3) | 0 |
| Neoplasms benign, malignant and unspecified (including cysts and polyps) | 1 (8.3) | 0 |
| Neoplasm progression | 1 (8.3) | 0 |
| Renal and urinary disorders | 1 (8.3) | 1 (50.0) |
| Acute kidney injury | 0 | 1 (50.0) |
| Urinary tract obstruction | 1 (8.3) | 0 |
| Respiratory, thoracic and mediastinal disorders | 1 (8.3) | 0 |
| Acute pulmonary oedema | 1 (8.3) | 0 |
| Vascular disorders | 1 (8.3) | 0 |
| Thrombophlebitis | 1 (8.3) | 0 |

AEs were coded by system organ class and preferred term using MedDRA v25.1.

MedDRA, Medical Dictionary for Regulatory Activities; SAE, serious adverse event.

SAEs with a fatal outcome occurred in 2/12 (17%) and 1/2 (50%) patients in the aztreonam-avibactam and BAT groups, respectively. In the aztreonam-avibactam group, one patient with HAP/VAP experienced multiple organ dysfunction syndrome and sepsis that resulted in death on study day 1 and another patient with BSI died from neoplasm progression on study day 31. In the BAT group, one patient with HAP/VAP experienced cardiac arrest and multiple organ dysfunction syndrome that resulted in death on study day 3.

AEs of special interest (liver disorders, *Clostridioides difficile*-associated diarrhoea and hypersensitivity/anaphylaxis) were identified by Standardized MedDRA Queries (SMQs). Five liver disorder AEs were reported in 2/12 (17%) patients in the aztreonam-avibactam group (0% in the BAT group): one cIAI patient had an AE of hepatic function abnormal, and one cUTI patient had AEs of increased alanine aminotransferase, aspartate aminotransferase, blood alkaline phosphatase and gamma-glutamyl transferase. These AEs were all non-severe. There were no confirmed Hy’s Law cases.

One patient each in the aztreonam-avibactam group (8%) and the BAT group (50%) experienced AEs in the hypersensitivity/anaphylaxis SMQs. In the aztreonam-avibactam group, a cIAI patient experienced an AE of moderate chest discomfort that was considered treatment-related and resolved without intervention. In the BAT group, an AE of cardiac arrest in a HAP/VAP patient was included in the hypersensitivity/anaphylaxis SMQs (as noted above, the AE was severe and resulted in death). No patients in either treatment group experienced AEs related to *C. difficile*-associated diarrhoea. The incidence of potentially clinically significant laboratory test abnormalities was low in both treatment groups, and there were no clinically meaningful findings in vital sign measurements, electrocardiograms and physical examination assessments.
